# Environmentally Friendly Flexible Strain Sensor from Waste Cotton Fabrics and Natural Rubber Latex

**DOI:** 10.3390/polym11030404

**Published:** 2019-03-01

**Authors:** Xinzhu Chen, Jing An, Guangming Cai, Jin Zhang, Wu Chen, Xiongwei Dong, Licheng Zhu, Bin Tang, Jinfeng Wang, Xungai Wang

**Affiliations:** 1 National Engineering Laboratory for Advanced Textile Processing and Clean Production, Wuhan Textile University, Wuhan 430073, China; cxz_wtu@163.com (X.C.); guangmingcai2006@163.com (G.C.); wuchen@wtu.edu.cn (W.C.); xwdong@wtu.edu.cn (X.D.); lichengz84@sina.com (L.Z.); xungai.wang@deakin.edu.au (X.W.); 2 Zhuhai College of Jilin University, School of Chemical Engineering and New Energy Materials, Zhuhai 519041, China; 15031@jluzh.com; 3 Deakin University, Institute for Frontier Materials, Geelong, VIC 3216, Australia; jin.zhang@deakin.edu.au

**Keywords:** natural rubber latex, cotton, recycling, carbonization, conductive fabric, strain monitoring

## Abstract

A green approach was successfully developed to fabricate flexible sensors by utilizing carbonized waste cotton fabrics in combination with natural rubber latex. Waste cotton fabrics were firstly carbonized by heat treatment in the nitrogen atmosphere before they were combined with natural rubber latex using three methods, i.e., vacuum bagging, negative pressure adsorption and drop coating. After impregnation with natural rubber, the carbonized cotton maintained the fabric structure and showed good conductivity. More importantly, the electric resistance of the textile composites changed with the tensile strain. The cyclic stretching-releasing tests indicated that the prepared wearable flexible strain sensors were sensitive to strain and stable under cyclic loading. The flexible strain sensor also demonstrated the capability of monitoring human finger and arm motion.

## 1. Introduction

Flexible sensors have attracted considerable interest for use in various applications, such as motion recording [1,2,3,4,5], health monitoring [6,7,8], heated garments [9] and sweat analysis [10], by virtue of their flexible, stretchable and wearable characteristics. Most of flexible sensors are composed of active conductive components and flexible substrates/matrix [11,12,13,14,15,16,17]. The most commonly used fabrication methods of flexible sensors include immersion [14], coating [12], printing [18] and molding [19]. During the immersion process, the flexible substrates were immersed (soaked or dipped) into the solution of active components such as graphene oxide (GO) and reduced graphene oxide (RGO). Zhang et al. prepared the strain sensors through immersing a polymer sponge into GO solution and then reducing GO by heat treatment [14]. Wang et al. fabricated a flexible electrically resistive-type strain sensor by immersing thermoplastic polyurethane (TPU) electrospun mats into RGO solution [1]. Larimi et al. prepared a sensor by soaking and infusing graphene nano-flakes into a rubber-like adhesive pad [3]. A highly sensitive and skin-like pressure sensor consisting of polydimethylsiloxane (PDMS) sheets and GO was also prepared [4]. Cai et al. produced flexible and wearable strain sensing fabrics by dipping fabrics in GO suspensions and then reducing GO nanosheets by NaBH_4_ solution. A coating method was also used to develop flexible sensors [20]. Liu et al. obtained flexible strain sensors by combining core-spun elastic yarn with conductive nanocomposite via a coating-drying process [12]. An all-fibre hybrid piezoelectric-enhanced triboelectric nanogenerator was fabricated by electrospinning silk fibroin and polyvinylidene difluoride (PVDF) nanofibers onto conductive fabrics [21]. Screen printing of silver nanoparticles on TPU substrate [22] and deposition of conductive components via vacuum filtering [13,23] have also been used in attempts to prepare flexible sensors. In a braiding approach, sensors were fabricated by braiding conductive fibers into a woven fabric, which was then covered with a polymer substrate [6,24]. The blending-molding approach, however, mixes the active conductive components with substrate materials by mechanical stirring or sonication treatment, and then pours the mixture into mold for various shapes. For example, TPU incorporating carbon black or carbon nanotubes was molded to form flexible composites [25]. Mixtures of PDMS elastomer and conductive nanomaterials (e.g., graphene) were also cured in shape for preparing flexible sensors [15,26]. In addition to PDMS, other polymer elastomers such as epoxy [7,8], and poly(glycerol sebacate) [2] have also been utilized for fabricating flexible sensors. Moreover, carbonized fabrics obtained from traditional textile fabrics such as woven silk and weft-knitted Modal textile were used as conductive components to prepare strain sensor and conductive textile by Zhang’s group [27,28]. Compared with graphene and carbon nanotubes (CNTs), the biomass-derived carbon-based materials exhibited many advantages, including scalability, low-cost and eco-friendly nature [29,30,31].

A large amount of textile waste was produced because of the boom of fast fashion after years of extraordinary growth of the fashion industry and because of rising living standards [32]. A circular economy evinces a strong preference for maintaining value, or even increasing the value of the recycled product (‘upcycling’). Some upcycling examples are to reuse waste textiles to reinforce composite materials [33,34]. As one of the most commonly used textile materials, cotton has gained intensive attention in upcycling because of its natural convolutions, the fact that a major component of cellulose as carbon precursor, and because of its numerous active hydroxyl groups [35]. Cotton fabric as a flexible natural material has unique features such as a porous structure, high surface area, lightweight, good flexibility and recoverable deformation [36,37]. Meanwhile, natural rubber latex (NRL) is an eco-friendly polymer elastomer, with a good combination of rigidity and elasticity. Fibrous materials and carbon black have been used to reinforce and further improve the mechanical properties of natural rubber [38,39,40]. Conductive rubber materials have been developed by incorporating conductive fillers (e.g., graphite and graphene). To the best of our knowledge, using natural rubber [41,42,43] to prepare flexible sensors was an under-researched area. The use of waste and recycled textiles is becoming significant to sustainability and the environment. Combining natural rubber and recycled fabrics, the fabrication of composites will contribute to waste management and sustainable development.

Herein, a facile, low-cost and environment-friendly method has been successfully developed to fabricate flexible strain sensors by impregnating carbonized cotton fabrics with NRL. The carbonization process was carried out by heating the waste cotton fabric in the nitrogen atmosphere. Three combination methods including vacuum bagging, negative pressure adsorption and drop coating were employed to combine carbonized fabrics and NRL, which significantly enhanced the ultimate strength and strain of carbonized fabric. The morphology and surface chemistry of the carbonized fabrics and textile composites were characterized. Vacuum bagging was shown to provide the optimized integration of carbonized fabric and NRL. The electromechanical performance and strain sensing properties of the obtained textile composites from vacuum bagging were investigated. The flexible strain sensor was also used to monitor human finger and arm motions.

## 2. Materials and Methods

### 2.1. Materials

**Figure 1:**
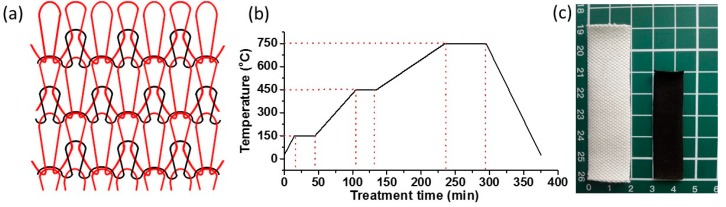
(**a**) Illustration of knitted structure of cotton fabric. (**b**) Heat treatment process of cotton fabrics. (**c**) Photograph and diagram of the cotton fabric before and after carbonization.

Waste knitted cotton fabrics from cardigans (212.7 g·m^−2^) with 143 wales (per 5 cm) and 48 courses (per 5 cm) were used in this research. The structure of knitted cotton fabric is illustrated in Figure 1a. Natural rubber latex was kindly provided by the Agricultural Product Processing Research Institute, Chinese Academy of Tropical Agricultural Sciences, Zhanjiang, China.

### 2.2. Carbonization of Cotton Fabric

Carbonization of waste cotton fabrics was performed in a tube furnace (TL1200, Nanjing BYT Instrument Technology Co., Ltd., Nanjing, China). Cotton fabrics were firstly placed into the center of a quartz tube in the furnace after purging the tube with nitrogen for 30 min. Figure 1b shows the heating process of the cotton fabrics. The samples were heated from room temperature to 150 °C and held at 150 °C for 30 min. The temperature continued to increase to 450 °C at a rate of 5 °C min^−1^ and maintained at 450 °C for 30 min. The samples were then heated to 750 °C at a rate of 3 °C min^−1^ and held at 750 °C for 60 min. Finally, the furnace was cooled down to room temperature to obtain the carbonized fabrics.

### 2.3. Textile Composites Manufacture

**Figure 2:**
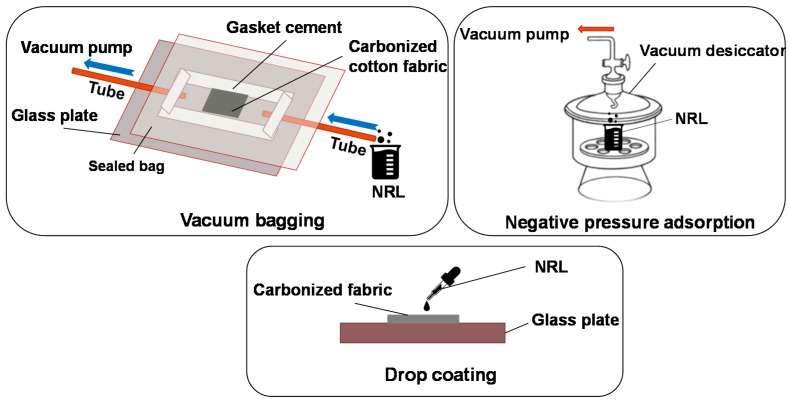
Schemes of Textile composites fabrication using three methods.

Three combination methods, i.e., vacuum bagging, negative pressure adsorption and drop coating, were used to impregnate carbonized fabrics with NRL (Figure 2). In the vacuum bagging method, carbonized fabrics were sealed in a plastic bag and the NRL was drawn into carbonized fabrics using a vacuum pump. The vacuuming time for each sample was 5 min. In terms of negative pressure adsorption method, carbonized fabrics were immersed directly into the NRL solution in a beaker which was located in a vacuum desiccator. Subsequently, the desiccator was vacuumed for 15 min. In the drop coating method, the NRL solution was dropped directly onto the carbonized fabrics. The impregnated carbonized fabrics were then cured at room temperature for 24 h.

### 2.4. Characterization

Scanning electron microscopy (SEM) was carried out using a JEOL JSM-6510LV scanning electron microscope (Tokyo, Japan). X-ray photoelectron spectroscopy (XPS) was performed on a Kratos XSAM800 XPS system with Kα source and a charge neutralizer (Manchester, UK). Raman analysis was performed on a Renishaw inVia Raman microscope system (Renishaw plc, Wotton-under-Edge, Gloucestershire, UK). A 50×/N.A. 0.75 objective and a 785-nm near-IR diode laser excitation source (500 mW, 10%) were used in all measurements. Raman spectra were recorded using a mounted CCD camera with integration time of 10 s by single scan. The mechanical properties were measured using an Instron Model 5566 Materials Testing System (Norwood, MA, USA). The tensile strength of specimens with a width of 15 mm at a gauge length of 40 mm was tested along the wale direction with a loading rate of 1 cm min^−1^. The changes in electric resistance of fabric samples at different strain levels were measured by a self-built fabric dynamic resistance tester. The tester is composed of a stretching device and a digital multimeter. For the stretching device, two jaws used for clamping fabric samples are set up on screw spindle driven by a motor which is controlled by software. The digital multimeter is connected to the jaws for resistance measurement of samples.

## 3. Results and Discussion

**Figure 3:**
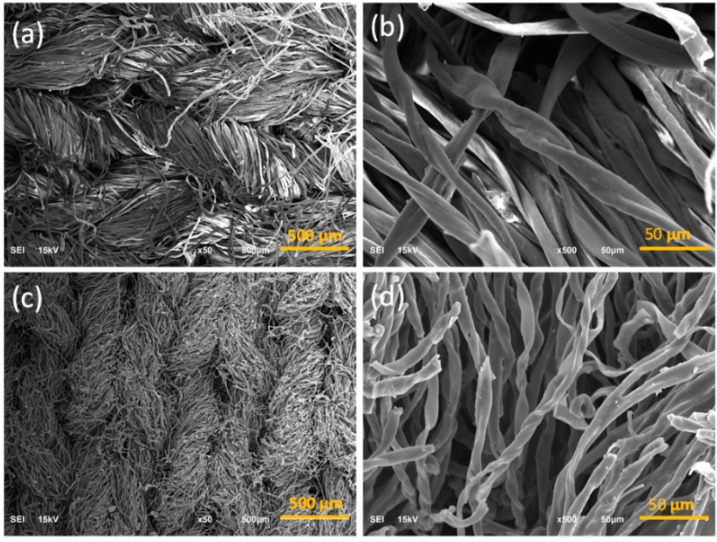
SEM images of the pristine cotton fabric (**a**,**b**) and carbonized fabric (**c**,**d**).

**Figure 4:**
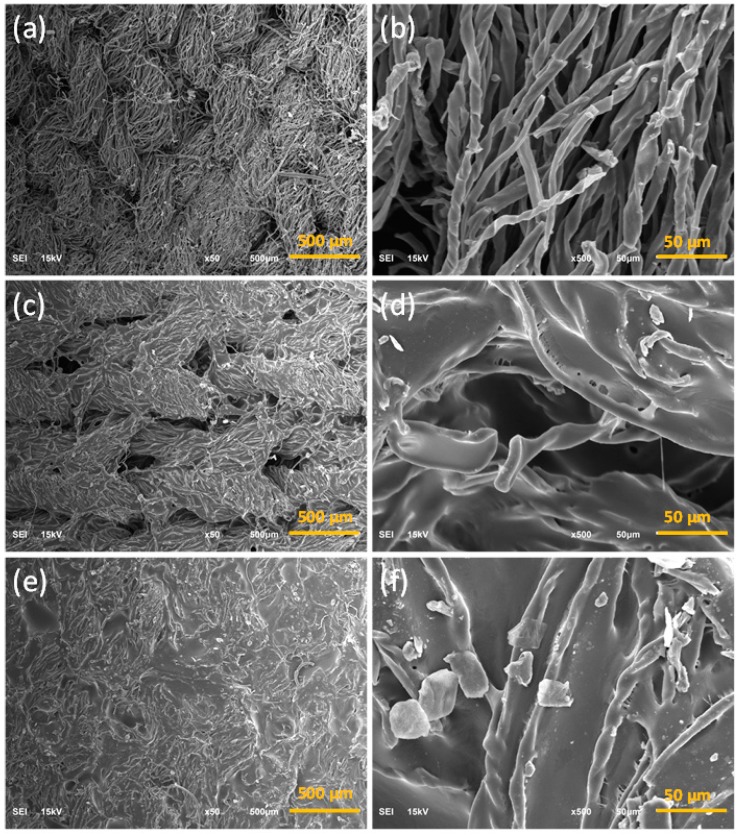
SEM images of the carbonized fabrics: (**a**,**b**) VB-CF, (**c**,**d**) NPA-CF and (**e**,**f**) DC-CF.

Cotton fabrics were converted into carbon fabrics by heating in the nitrogen atmosphere. Compared to the pristine fabric (PF), the surface area and the weight of carbonized fabric decreased by 47.3 ± 2.2% and 82.1 ± 1.4%, respectively (Figure 1c). Figure 3a,b show the SEM images of PF. The knit structure of the fabric and the convolutions of cotton fibers can be seen clearly. The carbonized cotton fabrics maintained the original knitted texture of PF with reduced volume after the carbonization process (Figure 3c,d). The heat treatment led to carbonization of cotton, but maintained the fabric and fiber structure. Figure 4a shows the morphology of obtained textile composites from the vacuum bagging (VB-CF) method that kept similar fabric structure as the pristine fabrics, with fibers separated from each other (Figure 4b). Textile composites obtained from the negative pressure adsorption (NPA-CF) method also displayed the notable knitted structure (Figure 4c). However, the carbonized fibers were crosslinked by NRL, which reduced the wearability of the obtained composites (Figure 4d). The wearability of the treated fabrics significantly affect the development of electronic textiles used for functional clothing. The composite specimen from drop coating (DC-CF) did not show obvious fabric structure (Figure 4e). Under high magnification, some fibers can be identified (Figure 4f), and all the fibers were bonded by NRL. Among the three coating methods, VB-CF retained the structural features of pristine cotton fabrics. All the fabrication methods maintained the fabric appearance, but significantly changed the microstructure of the obtained composite specimens.

**Figure 5:**
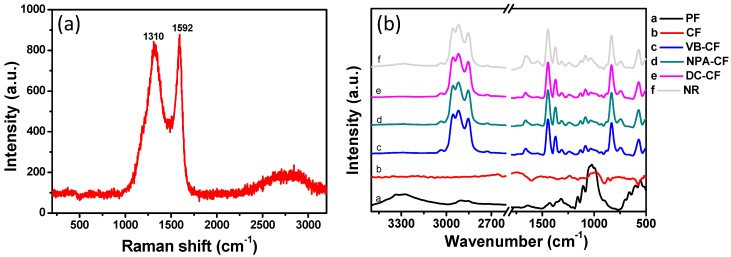
(**a**) Raman scattering spectrum of carbonized fabric. (**b**) FTIR spectra of the fabric samples.

Figure 5a presents the Raman scattering spectrum of the fabric after carbonization. Two remarkable scattering peaks at 1592 and 1310 cm^−1^ were observed, which are assigned to the G band (1592 cm^−1^) and D band (1310 cm^−1^) of graphite, respectively. The G band associates with the C–C bond in the highly ordered graphite lattice. The D band is related to the defects in graphite domain. A broad 2D band is also seen at around 2750 cm^−1^. The results indicate that the cotton was converted into carbon after the heat treatment. FTIR spectroscopy was utilized to analyze the surface chemistry of the samples (Figure 5b). The pristine cotton presents the bands at 3334, 2908, 1640, 1427, 1159 and 1107 cm^−1^, which could be assigned to OH stretching, C–H asymmetrical stretching, C=O stretching, symmetric CH_2_ bending, C–O–C asymmetrical stretching and C–O–H asymmetric stretching vibration modes, respectively (Curve a in Figure 5b) [44,45]. These bands are attributed to the characteristic bands of cellulose from cotton. No visible bands were observed in the FTIR spectrum of cotton after carbonization, implying that the active groups were eliminated in the process of heat treatment. After impregnation with natural rubber, new absorption bands appeared at 3285, 1653, 1541, 1445 and 1373 cm^−1^, which are attributed to N–H stretching (proteins), C=C stretching (Amide I), N–H in-plane bending or C–N stretching (Amide II), –CH_2_– deformation and –CH_3_ asymmetric deformation vibration modes of natural rubber, respectively (Figure 5b) [46]. These results demonstrate that natural rubber was coated onto the carbonized fabrics.

**Figure 6:**
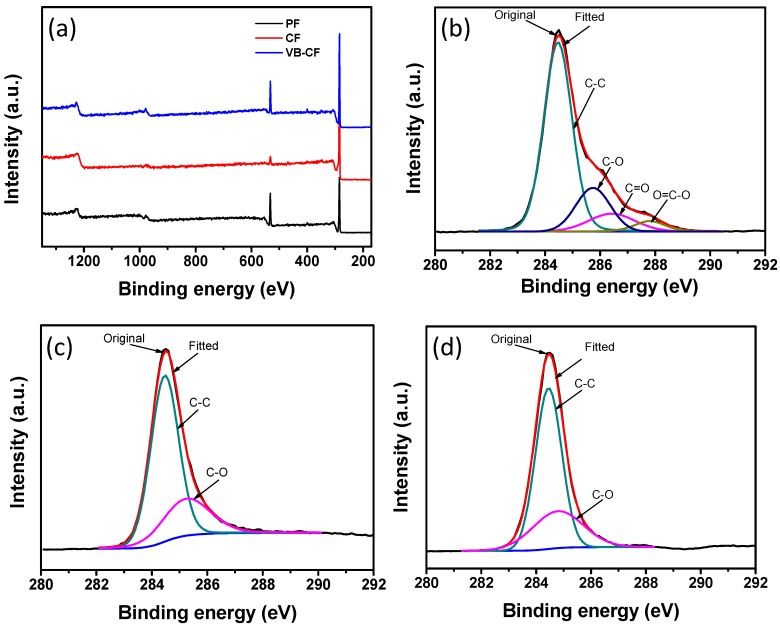
(**a**) XPS survey spectra of PF, CF and VB-CF. C1 XPS curves of (**b**) PF, (**c**) CF and (**d**) VB-CF.

Figure 6a shows the XPS spectra of pristine fabric, carbonized fabric and textile composites prepared by the vacuum bagging method. XPS peaks assigned to carbon and oxygen can be clearly seen in the spectrum of the cotton fabric. After carbonization, the intensity of the oxygen peak dramatically decreased, which reveals that carbonization eliminated the oxygen-containing groups on the fabrics. The oxygen peak increased again in the VB-CF spectrum due to the coating of natural rubber. C1 XPS spectra also confirmed that the oxygen species reduced and the carbon content increased after carbonization (Figure 6b,c). However, the carbon element on fabrics showed little change after the combination with natural rubber, which indicates the integration with NRL did not significantly change the surface chemistry of carbonized fabrics (Figure 6d).

The tensile tests were carried out to gain insights into the effect of combination of natural rubber on the mechanical properties of fabrics. Also, the relative resistance change (∆R/R_0_) of the specimens as a function of strain in the wale direction was evaluated, where R_0_ denotes the initial resistance value, and ∆R represents the real-time resistance (R) with strain subtracted by R_0_. The same type of samples from different batches showed the similar stress-strain curves with the same change trends (Figure S1, see Supplementary Materials) though there were some small differences among different batches, which may be due to the inherent features of recycled fabrics and natural rubber latex. Meanwhile, the stain-dependent relative resistance changes of the samples from different batches were also obtained (Figure S2, see Supplementary Materials). The samples from different batches showed nearly same curves of relative resistance changes, indicating that the sensing fabrics prepared by the present methods exhibited great reproducibility. To make the discussion on the change trends clear, the samples from the same batch (Batch 1) were chosen to investigate the mechanical and electric properties of the strain sensing sensors.

**Figure 7:**
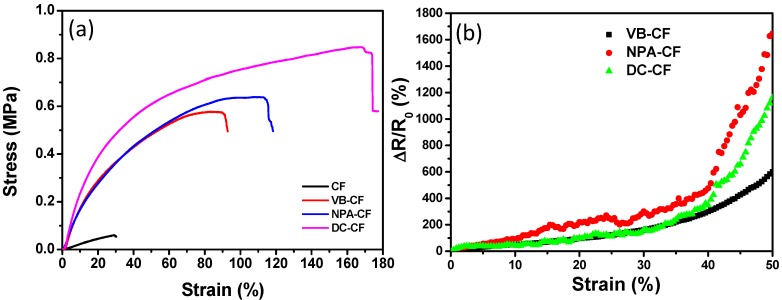
(**a**) Curves of stress *versus* strain corresponding to different fabric samples. (**b**) Relative resistance variation (∆R/R_0_) of different textile composites under tensile loading.

As shown in Figure 7a, the carbonization treatment significantly reduced the mechanical strength of the cotton fabrics. The ultimate stress of the carbonized fabric was 0.06 MPa with a maximum strain of 29.0%. The low mechanical property of carbonized fabrics limited their practical applications. Compared with carbonized fabric, the composite fabrics showed significantly increased breaking strength after impregnation with natural rubber. Among all the composites, DC-CF showed the highest ultimate stress of 0.85 MPa, which was followed by NPA-CF of 0.64 MPa and VB-CF of 0.58 MPa. The content of natural rubber in the VB-CF, NPA-CF and DC-CF was measured to be 77.6, 85.4 and 85.9 wt%, respectively. These results showed that the maximum strength of the obtained composites increased with the increasing content of natural rubber. The maximum strain values were 89.8% for VB-CF, 113.4% for NPA-CF and 168.6% for DC-CF. The higher content of natural rubber led to increased strain of the textile composites (Figure 7a). Compared with the carbonized fabrics, the breaking strength of composites (e.g., VB-CF) increased by 8.5 times and the corresponding strain by 2.1 times. The impregnation with natural rubber highly improved the mechanical properties of carbonized fabrics, which enables these fabrics to be used in wearable electronic devices.

**Figure 8:**
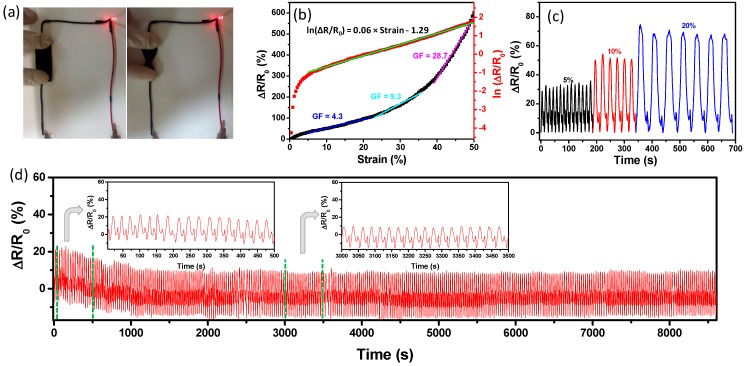
(**a**) Photographs of VB-CF wired with a light-emitting diode (LED) under original state and being twisted. (**b**) Curves for ∆R/R_0_ and corresponding ln(∆R/R_0_) of VB-CF under tensile loading. Purple line is the linear fitting line for the plot of ln(∆R/R_0_) versus loading of stretching. (**c**) ∆R/R_0_ curves of VB-CF under cyclic tensile strain of 5%, 10% and 20%. (**d**) The relative resistance variation of VB-CF under cyclic tensile strain of 5% for 320 cycles.

Figure 7b shows the plots of the ∆R/R_0_ of the specimens versus loading strain. The ∆R/R_0_ increased monotonously when the specimens were stretched in the range of 0–50%. The composite fabrics (VB-CF, NPA-CF and DC-CF) with high elasticity displayed sensitive response to strain, which demonstrates that the textile composites can be used as strain sensors with a wide stretchable range. For all three specimens, the resistance increased with the increasing strain, which may be caused by the disconnecting of partially carbonized fibers in the specimens. VB-CF showed the smoothest curve among all specimen types, which may be attributed to the effective combination of rubber and carbonized fabrics. NPA-CF and DC-CF are not suitable for straining sensing due to the unstable resistance changes under increasing stretching. The strain sensor is conductive, flexible and durable. As can be seen from Figure 8a, a light-emitting diode (LED) contented with the composite fabric (VB-CF) was lit up. The twisted fabric still displayed excellent conductivity, wiring the lit LED. By virtue of the stable response to strain, VB-CF was further tested and analyzed for sensing performance. It was found that the relative resistance change with an increase in loading strain did not fit linear relationship (Figure 8b). The gauge factor (GF) values for the resistance changes of VB-CF were calculated in three stages. The first stage of the ∆R/R_0_ curve has a small GF of 4.3. And then the GF of the sensing fabric increased to 9.3 at the second stage. The largest GF values was obtained as 28.7, which is comparable to that in the previously reported literature [27,47]. Although the ∆R/R_0_ curve could provide the relative resistance changes at the certain stretching, the relationship between ∆R/R_0_ and loading strain is not clear via the description of GF. The natural logarithm of ∆R/R_0_ (ln(∆R/R_0_)) vs. loading strain was plotted in Figure 8b. Two phases can be seen in the plot of ln(∆R/R_0_) as a function of loading strain. In the first phase, the ln(∆R/R_0_) increased dramatically as the loading increased in a small strain range (<4.5%). With an increase in loading strain (4.5–50 %), the ln(∆R/R_0_) increased linearly in the second phase. The relationship between ln(∆R/R_0_) and strain could be obtained by linear fitting, which facilitates the sensing applications of composite fabrics. It is suggested that the resistance changes could be attributed to the deformation of fabric and the disconnection of conductive components in fabric. Figure 8c shows the relative resistance change of VB-CF with 5%, 10% and 20% strain under stretching-releasing cycles. It can be seen that the ∆R/R_0_ increased with the increasing of strain. The resistance changed slightly under the cyclic stretching and releasing, suggesting the sensing performance was fairly stable. To further study the strain sensing performance, a stretching-releasing of 5% strain was carried out under 320 cycles at frequencies of 0.037 Hz (Figure 8d). There were hysteresis of relative resistance changes at the initial stage of the strain cycles (less than 40 cycles). The relative resistance drifted downward slightly (less than 40 cycles) and then became steady without visible hysteresis (more than 40 cycles). The data indicates that the flexible strain sensor tended to be stable with the cyclic loading of strain. The initial hysteresis could be due to the creep behavior of natural rubber [48]. The interaction between carbonized fibers and coated rubber could also cause the hysteresis at the beginning of cyclic strain loading [49,50,51]. In addition, the possible structure changes of the carbonized fabrics may be another reason for hysteresis [52]. The insets in Figure 8d display the specific curves of relative resistance changes versus strain in different stages in the cyclic stretching-releasing process. It can be found that the hysteresis of relative resistance change was low, even for the initial strain loading cycles.

The conductive element in the present research was from the carbonization of recycled cotton with a lower cost. The polymer matrix was NRL, with a lower cost than the processed rubber products. Thus, the present fabrication method is supposed to be low-cost. Moreover, no organic solvents were required in the preparation process. The fabrication approach of the composite fabrics needed only two steps, carbonization of cotton and combination with natural rubber, which is an easy-to-implement strategy. It should be noted that the polymer matrix for composite fabric was unvulcanized natural rubber. The stability and durability may be further improved by vulcanization of rubber matrix in composites.

**Figure 9:**
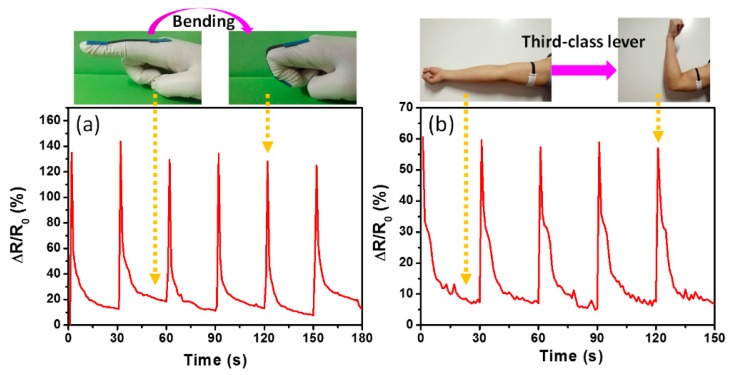
Monitoring pattern of (**a**) finger bending and (**b**) contracting of dizzy biceps during third-class lever by using VB-CF.

The as-prepared textile composites were also used as wearable devices to monitor real-time human motion. Figure 9a shows the pattern of ∆R/R_0_ of VB-CF during finger bending. The resistance increased when the finger was bent. The peaks corresponded to the bent finger and the trough of wave was attributed to the flat finger, which suggests that the bending motion of a finger can be monitored by the sensing composites. The muscle contraction of the human body can also be monitored. Figure 9b shows the relative resistance changes of VB-CF corresponding to the contracting of dizzy biceps during the third-class lever. The dizzy biceps were relaxed when the forearm was placed flat, which leads to around 7.5 of the ∆R/R_0_ at the trough of waves. The ∆R/R_0_ significantly increased to 57 when the dizzy biceps contracted from the third-class lever of forearm, which has a similar trend as the resistance changes for finger bending. However, the profile of ∆R/R_0_ curves for finger bending and muscle contraction shows a different amplitude and shape, which implies that the sensing composites could be used to distinguish body actions.

## 4. Conclusions

In summary, recycled cotton fabric was carbonized by heat treatment and then combined with natural rubber latex (NRL). The obtained textile composites can be used as wearable strain sensors. The carbonized fabric sustained the original knit structure of the cotton fabrics. The bonding of NRL visibly enhanced the mechanical properties of carbonized fabrics. The relative resistance of the obtained composite fabric increased as the strain increased, demonstrating its strain sensing ability. This flexible strain sensing textile composite showed stable resistance changes under repeated stretch-release cycles. Finger bending and muscle contraction were monitored using the fabricated sensing fabrics, which testifies the effective sensing performance of the composites. No organic solvents were required during the preparation flexible strain sensors based on the recycled textile and natural rubber, within the scope of green chemistry. The utilization of the waste textile not only reduces the cost of the functional composites, but also facilitates environmental protection.

## Supplementary Materials

The following are available online at https://www.mdpi.com/2073-4360/11/3/404/s1, Figure S1: Curves of stress versus strain corresponding to the fabric samples from different batches, Figure S2: Relative resistance variation (ΔR/R0) under tensile loading for textile composites from different batches.
