# Supplementary material for: Environmentally Friendly Flexible Strain Sensor from Waste Cotton Fabrics and Natural Rubber Latex
Source: Polymers (Basel). 2019 Mar 1;11(3):404. doi: 10.3390/polym11030404 (PMC6473477; doi:10.3390/polym11030404)
Supplement: Supplementary file 1 [file polymers-11-00404-s001.pdf]

Supplementary Materials

Environmentally Friendly Flexible Strain Sensor from Waste Cotton Fabrics and Natural Rubber Latex

Xinzhu Chen [1], Jing An [2], Guangming Cai [1], Jin Zhang [3], Wu Chen [1], Xiongwei Dong [1], Licheng Zhu [1], Bin Tang [1,3,*], Jinfeng Wang [1,3,*] and Xungai Wang [1,3]

[1] National Engineering Laboratory for Advanced Textile Processing and Clean Production, Wuhan Textile University, Wuhan 430073, China; cxz_wtu@163.com (X.C.); guangmingcai2006@163.com (G.C.); wuchen@wtu.edu.cn (W.C.); xwdong@wtu.edu.cn (X.D.); lichengz84@sina.com (L.Z.); xungai.wang@deakin.edu.au (X.W.)

[2] Zhuhai College of Jilin University, School of Chemical Engineering and New Energy Materials, Zhuhai 519041, China; 15031@jluzh.com

[3] Deakin University, Institute for Frontier Materials, Geelong, VIC 3216, Australia; jin.zhang@deakin.edu.au

* Correspondence: bin.tang@deakin.edu.au (B.T.); jinfeng.wang@deakin.edu.au (J.W.); Tel.: +61-3-5227-2012 (B.T. & J.W.)

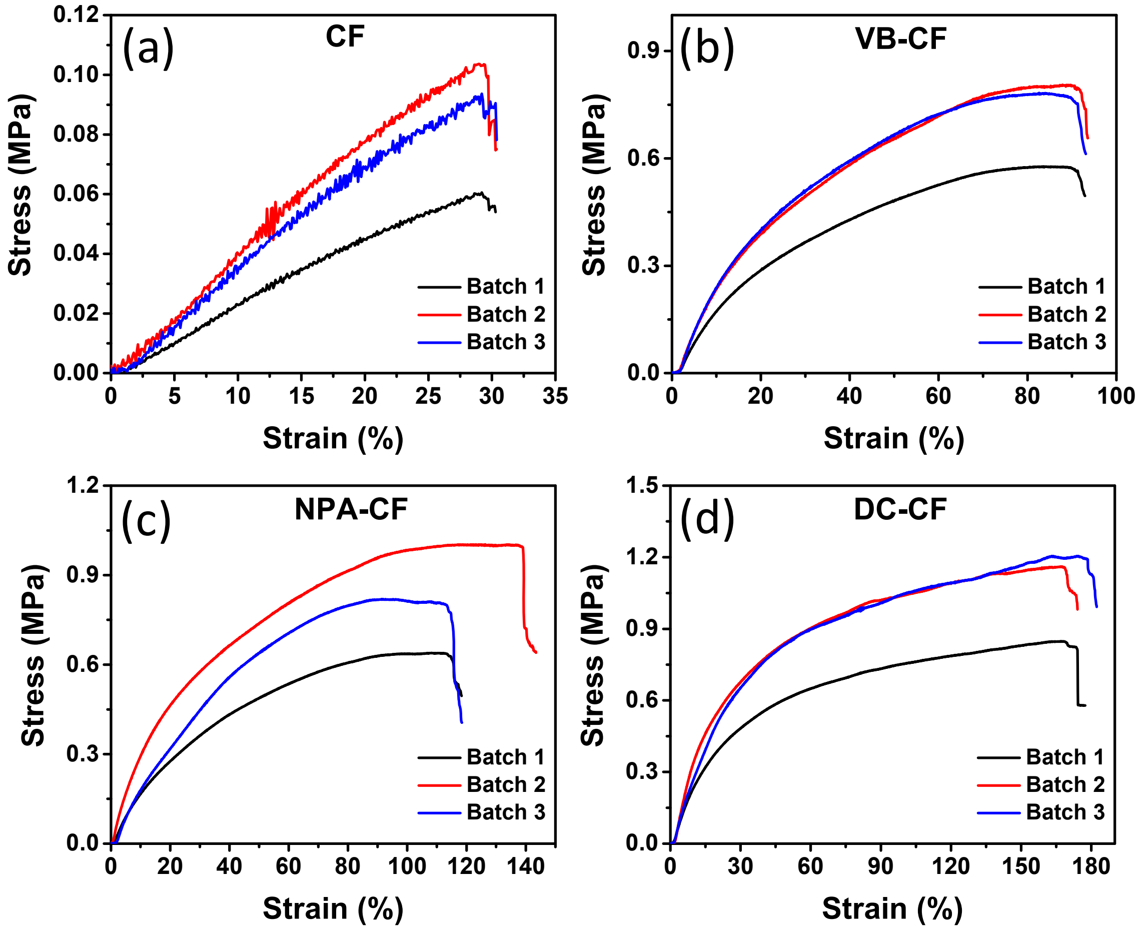

Figure S1. Curves of stress versus strain corresponding to the fabric samples from different batches.

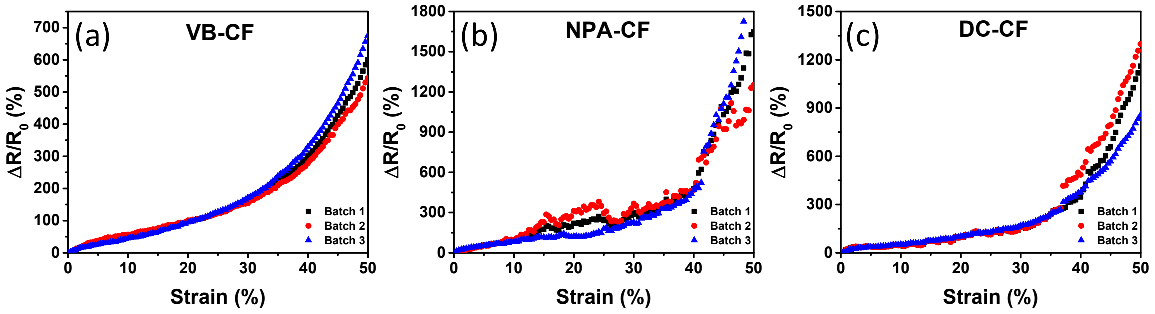

Figure S2. Relative resistance variation ($\Delta R/R_0$) under tensile loading for textile composites from different batches.
